# A (metaphorical) moment for RNA-based biotechnology? Current metaphors for RNA limit development of and public communication about RNA-based technologies

**DOI:** 10.1038/s44319-024-00200-y

**Published:** 2024-07-08

**Authors:** Erika A Szymanski, Daniel Schindler

**Affiliations:** 1 Department of English, Colorado State University, Fort Collins, CO 80521 USA; 2 Max-Planck-Institute for Terrestrial Microbiology, 35043 Marburg, Germany; 3 Center for Synthetic Microbiology, Philipps-University Marburg, Marburg, Germany

**Keywords:** History & Philosophy of Science, RNA Biology, Science Policy & Publishing

## Abstract

Technical and societal hurdles are both likely to hinder the development of RNA biotechnologies. The use of current metaphors to describe RNA and its function could be an important factor for a common challenge.

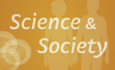

Who, other than biologists, thought about—or was even aware of—the existence of RNA before the summer of 2020? A few newspaper articles about the AIDS epidemic in the 1980s and 1990s mentioned HIV’s RNA genome but largely as a scientific sidebar to the main story about gay rights. RNA interference (RNAi) for “silencing” disease-causing genes was briefly exciting and then rapidly less so in the late aughts, in a bubble that only dedicated science news-followers would have sighted. A series of RNA-related Nobel prizes have ensured that DNA’s poor relative makes an occasional appearance in the news. But the advent of mRNA vaccines in the wake of the COVID-19 pandemic was likely the first time that non-experts had a compelling reason to know and care about RNA—or to think about how RNA-based technologies mattered to them in a personal register. That unbothered ignorance of RNA and its functions will not last long. As a synthetic biologist and a rhetorician of science, we perceive that applying engineering approaches to RNA calls for new scientific and societal communication strategies alongside technology innovation—and for interdisciplinary research that accounts for how these interact.

“the advent of mRNA vaccines in the wake of the COVID-19 pandemic was likely the first time that non-experts had a compelling reason to know and care about RNA.”

## RNA, the next great challenge in biology

RNA-based biotechnologies seem poised for a surge, and not solely because of the success of mRNA vaccines against SARS-CoV2. In addition to renewed interest in RNAi therapeutics, RNAs will have numerous future applications to produce recombinant proteins, biosynthesis pathways, or antigens (Hastings and Krainer, 2023). In an underappreciated role, small non-coding RNAs are also central to much-discussed CRISPR/Cas technologies.

The US National Academies of Sciences, Engineering, and Medicine (NASEM) just published a report, *Charting the Future* (2024), that identifies epitranscriptomics—the comprehensive study of cellular RNA modifications—as the next big challenge for molecular biology, advocating a grand RNA sequencing effort similar to the Human Genome Project. That effort, the report suggests, should support innovations not only in biomedicine but across industry, defense, agriculture, and synthetic biology. And while the NASEM report focuses on sequencing epitranscriptomic modifications in mRNA, tRNA, and rRNA, plenty of those advances are likely to involve various small “non-coding” RNAs in diverse catalytic, regulatory, and storage roles. RNA biologists have for several decades been populating a menagerie of RNA classes that have nothing to do with the now clearly outdated Central Dogma that “DNA makes RNA makes protein” or its implication that RNA is a mere messenger—a role played by only about 5% of a cell’s total RNA (Alberts et al, 2002). These other RNAs are likely to prove rich terrain for biotechnology.

Developing RNA-based biotechnologies comes with myriad challenges, including a lack of specific tools and the need to communicate with broader audiences in the near-absence of public conversation or knowledge about biology’s second-fiddle nucleic acid. Although those technical and “outreach” challenges are generally seen as separate, they overlap in that both revolve around communication. Both technical and societal limitations of working with RNA are grounded in genetic text metaphors that analogize DNA, and by extension RNA, to language. Those metaphors are ubiquitously applied to nucleic acids inside and beyond science, and increasingly limit accommodating and understanding the nuances of how nucleic acids are now understood to function. Because these challenges share a common root, they present a common opportunity to improve discourse tools for handling nucleic acids in the lab and for handling them in public communication as a single, integrated concern. Both call for innovation in metaphors—the simultaneously conceptual and communicative tools we all use to make sense of how phenomena relate to each other.

“Both technical and societal limitations of working with RNA are grounded in genetic text metaphors that analogize DNA, and by extension RNA, to language.”

By recognizing how the technical and the social connect, scientists and communicators might build strategies for biotechnologies and for communicating about biotechnologies in ways that align with scientific work, that expand collective capacity for exploring what those biotechnologies can and should do. Our argument is that scientists and society at large need to rethink genetic text metaphors to achieve the goals of biomedical research and biotechnology development more broadly.

## Metaphors as biotechnology tools

Technological innovation for engineering RNAs requires communication innovation because technologies for working with nucleic acids are built on metaphors. Or, rather, they’re built on *a* metaphor: the genetic code and the convention of representing complex three-dimensional biological entities called DNA and RNA in Roman alphabet letters such that molecules can be written as lines of text in interfaces designed to handle human language.

Lily Kay (2000), a historian of science, observed that the metaphor of the “genetic code” was a mistake; the relationship between DNA and protein sequence is, technically, a table of correlations. No matter: it’s still biology’s most prominent example of how metaphors underwrite scientific developments. Scientists read (*sequence*), write (*synthesize*), and decode (*annotate*) DNA. Computer-aided DNA design tools rely on language models to assemble and rearrange bits of text that will later be synthesized as molecules (Szymanski and Scher, 2019). The same algorithms used to search and structure human language are employed to search and structure DNA-sequencing data (Stevens, 2016). CRISPR/Cas is described as a DNA *editing* tool. Digital data is encoded into DNA for long-term storage by translating binary code into genetic code. Working with nucleic acids without envisioning them in terms of text is virtually impossible.

The weight and power of those metaphors focus sharply on RNA’s textual functions. Simultaneously, they leave the field bereft of tools to understand RNA features that are not well accounted for by text metaphors: tertiary or three-dimensional and quaternary or interactional structure, “non-coding” regulatory roles that depend on structure rather than sequence, and the extra-textual or epitranscriptomic modifications at the center of the NASEM report. Mapping RNA structures remains mostly indirect, as crystallography and cryoelectron microscopy are primarily used to determine structures of small or truncated RNAs as components of protein complexes. Flexible, single-stranded RNA forms complex three-dimensional structures, yet sequencing technologies rely on destroying those structures, forcing them into linearity to deliver a one-dimensional textual view. Reasonably robust algorithms can infer flat two-dimensional structures from those sequences, but tools for reconstructing three-dimensional structures are unreliable at best.

Despite some progress, the number of available RNA structures remains two orders of magnitude lower than the number of available protein structures (Das, 2021). Interactions between DNA structure and its informational properties are similarly understudied—a gap that is now also limiting the development of larger-scale DNA engineering. For both RNA and DNA, that which lies outside the dominant metaphors that guide how we—literally—look at these molecules is, more often than not, simply not captured by technologies for observing and manipulating them. Certainly, studying RNA in three dimensions is technically challenging. But the same could be said about studying protein structure, the importance of which has long been recognized. And where abundant protein crystal structures have enabled highly predictive machine-learning tools such as AlphaFold, the cognate RNAfold can generate only two-dimensional structures (Lorenz et al, 2011).

Limitations that apply to communicating about RNA among non-specialist audiences are structurally similar. Broader societal conversations about RNA barely exist. Where they do, they rely on characterizing RNA as a messenger in ways that will do nothing at all to help scaffold discussions about RNA’s regulatory or catalytic functions. Indeed, the existing science communication toolkit for genetics and genomics is all too likely to hinder rather than help as genetic biotechnologies diversify.

“the existing science communication toolkit for genetics and genomics is all too likely to hinder rather than help as genetic biotechnologies diversify.”

## Innovations in RNA metaphors

Meanwhile, RNA biologists have been innovating loads of metaphors to make sense of novel RNA classes and functionalities. Beyond being a messenger, RNA can be a monster, a building material, a machine, a fuel, a scaffold, a template, an expense, a fossil, a switch, a parasite, a sponge, a signal, a decoy, a guide, reporter, housekeeper, knife, puzzle piece, address, the “world” from which all life may have originated, and so on. Trying to make a comprehensive list is almost pointless, because anything we write today will be out of date by the time you read this tomorrow. That array of metaphors should be a rich resource for further innovation in how to engineer those RNA species and how to explain them to non-specialists. Certainly, they all relate to everyday experiences in ways that make their meanings widely accessible. In technical practice, however—and this goes for communicating technical work with wider audiences, too—they largely follow the same pattern set by the genetic code.

“RNA biologists have been innovating loads of metaphors to make sense of novel RNA classes and functionalities.”

RNA-specific metaphors continue to distinguish information, described in textual terms, from structure, described in terms of shapes. The language of the RNA biology literature also reinforces that other-than-messenger RNA functions are outliers—even as biological evidence suggests otherwise. RNA is coding or non-coding. Structures involve Watson-Crick (WC) or non-WC base pairing, or base pairing is described as standard or canonical versus non-standard or non-canonical. When RNA is something other than a linear copy of DNA, it is described as surprising or exceptional.

Moreover, the metaphors and conceptual frameworks underpinning RNA biology are segregated by subfield. Origins of life researchers tend to focus on its ability to reproduce and evolve. RNA origami and nanotechnology researchers write about it as a building material, focusing on its structural properties. Cell biologists write about RNA either in textual terms or as alternatives to the general textual rule. Synthetic biologists investigate RNA modularity toward standardizing programmable tools, for example, in protein-RNA chimera antibiotics (Popella et al, 2021).

Robust research in rhetoric and linguistics attests that mixing metaphors isn’t a barrier to comprehension; readers and listeners seem to have no trouble relating to a researcher who was left high and dry by her colleagues and so had to take center stage at a workshop and nailed it, even though she was supposed to be merely holding down the fort. On the contrary, mixing metaphors can account for more varied and nuanced detail than can be contained in a single metaphor, mitigating at least some of the blind spots of each. But what the current mishmash doesn’t do is suggest a ready strategy for developing RNA-centric tools that account for complex structures; they don’t integrate structure-function relationships across the sequence and other structural scales relevant to more-than-messenger RNA.

Because they are unevenly distributed across subfields, current metaphors also warn of a potential hazard in how RNA studies are evolving. Metaphors are not merely descriptive; they are also construction tools. They configure what we expect their targets to be like, what we expect them to be able to do, and, therefore, how we work with them, such that research objects are iteratively remade to better fit the metaphors applied to them. As subfields work through distinct metaphors, they may construct different versions or visions of RNA, including model systems and findings that may not easily cross over to other areas. That lack of coherence is a bigger problem for algorithms and other technical infrastructures, where coherent ontologies must ensure that individual tools can be linked in data-processing pipelines.

“Metaphors are not merely descriptive; they are also construction tools.”

An epitranscriptomics project, of the kind the recent NASEM report advocates, would no doubt be a tide that raises all RNA-studying boats. It may simultaneously bias generations of research and biotechnology against understanding and using small “non-coding” RNAs, if the infrastructure it sponsors is built for mRNA. That will especially be the case if advanced sequencing technologies continue to rely on features that uniquely characterize mRNAs, such as their poly-A tail. Specific protocols to detect and sequence modifications specifically in non-coding RNA also merit attention, for example, for how modifications likely modulate the stability of catalytic and regulatory RNA molecules (Watkins et al, 2022).

Moselio Schaechter (2007), distinguished emeritus professor of biology at Tufts, has argued that we are experiencing a third “golden age” of microbiology because DNA sequencing has unified a field that had previously fractured along topical and disciplinary lines. Before sequencing became a standard tool, microbial ecologists, medical microbiologists, cell and molecular biologists, and so on employed separate vocabularies and worked in separate circles. Having a common connection, a powerful common method and a shared vocabulary, has enabled integrations and synergies that weren’t possible for most of Schaecter’s career. While inferring causality would be going too far, it’s notable that this integrative age has also led to a “microbial moment” (Paxson and Helmreich, 2014) that has had *everyone*, broadly, talking about the value of microbial life. Rethinking tools for RNA seems to be an opportunity for the same kind of work.

## Interdisciplinarity is required for inclusive language

Current conceptual and discursive tools aren’t adept for RNA. Developing better ones needs to be a priority as part of developing improved technical tools. At the same time, the range of what has been accomplished by analogizing DNA to human and computer languages is quite incredible—no doubt in part because the range of what humans know how to do with human and computer languages is similarly impressive. What’s peculiar about the present state of molecular biology is just how little of that range is being explored. Current interpretations of the genetic code metaphor are lodged in a way of understanding communication that dates to early twentieth-century cybernetic theory. In light of how many other theoretical and disciplinary approaches to communication and language populate humanities and social sciences, this tradition seems bizarrely impoverished. A necessary response, we think, lies in coordinated consortia that can enroll researchers with varied expertise, including social scientists and humanities scholars.

Scholarship in literature and in rhetoric, for example, describes how information is always materially embodied in ways that matter to how communication instruments make meaning in context (Hayles, 1999). Rhetoricians apply ecological frameworks to parsing how contextual dependencies shape interactions among texts and readers (McGreavy et al, 2018). Distinguishing between information content in a cybernetic sense—which has only ever applied imperfectly to biology—and meaning-making in a rhetorical sense suggests thinking about function as constituted between a text and its “reader”. Doing so suggests an inversion of how structure and sequence are typically described, wherein structural relationships are clearly “primary” and sequence is constrained by structure. Leveraging the varied expertise of scholars who specialize in thinking about the structure and function of other kinds of texts might inspire additional avenues for adapting the existing slate of metaphors to better account for RNA, and for more of the complexities of how DNA is now understood to function, too.

Building capacity for RNA can’t just supplant or avoid the existing landscape of metaphors, nor should it—they’re incredibly useful—but we can expand the available options. Metaphors are useful precisely because they’re partial and imperfect. They also do the most to inspire additional approaches when they remain visible and “alive”—that is, when we can recognize that we’re using them.

## Conclusions for science communication in the sociotechnical context

Social studies of science demonstrate that the metaphors that underpin DNA sequencing and synthesis matter to wider societal concerns in how they structure biotechnology applications. At a societal communication level, diverse and potentially conflicting metaphors are just fine; again, humans adeptly piece together the respective functions of inconsistent metaphors applied to a single target. What is less than ideal, however, is a communication ecosystem in which popular language for RNA doesn’t line up with scientific language, such that detailed multi-stakeholder conversations about what can and can’t be done with RNA require a lot of experts saying “yes, but you see, it doesn’t really work like that.”

The history of communication around the Human Genome Project demonstrates how metaphors used deliberately by scientists can shape popular science communication—including “promotional metaphors” (Nelkin, 1994) designed to bolster political support. The field’s marketing panache have led many historians and sociologists to call genomics a promissory science—not because underinformed audiences misunderstood experts or jumped to unwarranted conclusions, but because scientists themselves made grand promises about what their work should achieve. “Book of life” and similar metaphors suggested that sequencing the human genome would almost automatically lead to understanding how humans work, and to developing a bevy of new therapeutics. They were useful for advertising, not for guiding the science itself, but extended from genetic text metaphors that were central to the technical details of genome sequencing—and that, we could say, misled scientists, too. Promotional metaphors appear to have helped accomplish what they were designed to do, but they were designed to advertise, not to build capacity for serious societal conversations about how scientific advances might become practical biotechnologies.

Assessing how ill-advised metaphors affect non-specialists’ understanding of and feelings about genetics and genomics is fraught. Cognitive linguistics experiments have evidenced that single metaphorical words can matter to how broad audiences develop opinions about whether, for example, crime is a public health problem demanding social reform versus an aggressor demanding stronger law enforcement (Thibodeau and Boroditsky, 2013). No such experiments have been conducted to assess the effects of potentially misleading metaphors in genetics and genomics. Yet qualitative studies demonstrate that context is important in how non-specialists interpret scientifically inaccurate metaphors, such as calling the human genome a blueprint, both through the prior beliefs and knowledge that audiences bring with them (Condit, 1999) and the context in which those metaphors themselves appear (Condit et al, 2001).

In other words, the audience’s understandings are *shaped* by metaphors, not controlled by them. Readers can and often do interpret deterministic metaphors with more nuance than experts might fear (Condit, 1999; Condit et al, 2001). Simultaneously, the available evidence, including the shape of popular science communication and biotechnology marketing itself, indicates that those metaphors can also enable misunderstandings that would be better to avoid entirely—a particular danger when direct-to-consumer genome sequencing services such as *23andMe* are reinforcing the idea that DNA can reveal who you really are. We need metaphors that enable more sophisticated understandings, and more sophisticated conversations, even if using better metaphors won’t magically produce enlightened publics.

“We need metaphors that enable more sophisticated understandings, and more sophisticated conversations.”

When the primary purpose of science communication is understood as delivering information, metaphors seem important principally as a strategy to make complex phenomena understandable. But if science communication is understood as a matter of enabling conversation, then metaphors need to be able to facilitate and underpin the way that numerous groups understand and discuss these technologies. Extant metaphors do little to support any kind of conversation at all beyond textbook explanations of the Central Dogma. Anecdotally—anecdotally, because RNA hasn’t been anyone’s focus in examining COVID-related communication—numerous news stories about mRNA vaccines have inaccurately or incompletely characterized RNA, often relying heavily on the Central Dogma that “DNA makes RNA makes protein.”

Ostensibly, the goal of biomedical and other biotechnical research is to improve health and well-being. But what does that mean in practice? Given the myriad interests, perspectives, and ways of knowing involved in answering that question, the answer can’t just come from scientists, pharma companies, and other stakeholders who fund the research. We need the societal capacity to discuss what RNA-based biotechnologies can do, particularly as they afford biomedical interventions that raise a distinct set of concerns in comparison to DNA-based gene therapy.

“We need the societal capacity to discuss what RNA-based biotechnologies can do, particularly as they afford biomedical interventions that raise a distinct set of concerns in comparison to DNA-based gene therapy.”

The relative lack of prior attention in and beyond biology communities, specifically to RNA biotechnologies, and to RNA as more than a messenger, is an opportunity to build new infrastructures. We’ll all be in a better position to achieve our collective goals if communication tools are treated as more than an afterthought.

### Supplementary information

Peer Review File
