# Supplementary material for: A (metaphorical) moment for RNA-based biotechnology? Current metaphors for RNA limit development of and public communication about RNA-based technologies
Source: EMBO Rep. 2024 Jul 8;25(8):3182–6. doi: 10.1038/s44319-024-00200-y (PMC11315921; doi:10.1038/s44319-024-00200-y)
Supplement: Supplementary file 1 — Peer Review File [file 44319_2024_200_MOESM1_ESM.pdf]

EMBO reports

A (metaphorical) moment for RNA-based biotechnology?

Erika Szymanski and Daniel Schindler

Corresponding author(s): Erika Szymanski (erika.szymanski@colostate.edu)

Review Timeline:

Submission Date:	25th Apr 24
Editorial Decision:	7th May 24
Revision Received:	31st May 24
Accepted:	27th Jun 24

Editor: Holger Breithaupt

Transaction Report: This article was editorially reviewed with board member advice.
